# Modeling interactions between brain function, diet adherence behaviors, and weight loss success

**DOI:** 10.1002/osp4.403

**Published:** 2020-02-25

**Authors:** Amanda N. Szabo‐Reed, Laura E. Martin, Jinxiang Hu, Hung‐Wen Yeh, Joshua Powell, Rebecca J. Lepping, Trisha M. Patrician, Florance J. Breslin, Joseph E. Donnelly, Cary R. Savage

**Affiliations:** ^1^ Department of Internal Medicine University of Kansas Medical Center Kansas City Kansas; ^2^ Department of Population Health Health University of Kansas Medical Center Kansas City Kansas; ^3^ Hoglund Brain Imaging Center University of Kansas Medical Center Kansas City Kansas; ^4^ Department of Biostatistics University of Kansas Medical Center Kansas City Kansas; ^5^ Graduate School of Social Work University of Denver Denver Colorado; ^6^ Center for Brain, Biology and Behavior University of Nebraska‐Lincoln Lincoln Nebraska

**Keywords:** behaviors, obesity, prefrontal cortex, weight loss

## Abstract

**Introduction:**

Obesity is linked to altered activation in reward and control brain circuitry; however, the associated brain activity related to successful or unsuccessful weight loss (WL) is unclear.

**Methods:**

Adults with obesity (N = 75) completed a baseline functional magnetic resonance imaging (fMRI) scan before entering a WL intervention (ie,3‐month diet and physical activity [PA] program). We conducted an exploratory analysis to identify the contributions of baseline brain activation, adherence behavior patterns, and the associated connections to WL at the conclusion of a 3‐month WL intervention. Food cue‐reactivity brain regions were functionally identified using fMRI to index brain activation to food vs nonfood cues. Food consumption, PA, and class attendance were collected weekly during the 3‐month intervention.

**Results:**

The left middle frontal gyrus (L‐MFG, BA 46) and right middle frontal gyrus (R‐MFG; BA 9) were positively activated when viewing food compared with nonfood images. Structural equation modeling with bootstrapping was used to investigate a hypothesized path model and revealed the following significant paths: (1) attendance to 3‐month WL, (2) R‐MFG to attendance, and (3) indirect effects of R‐MFG through attendance on WL.

**Conclusion:**

Findings suggest that brain activation to appetitive food cues predicts future WL through mediating session attendance, diet, and PA. This study contributes to the growing evidence of the importance of food cue reactivity and self‐regulation brain regions and their impact on WL outcomes.

## 1 INTRODUCTION

Obesity is a complex medical and behavioral problem that can be positively impacted by weight management interventions, including diet and exercise.^1^, ^2^, ^3^, ^4^ However, the underlying cognitive and brain function factors associated with weight gain and loss remain poorly understood. Neuroimaging has been used to examine the underlying neural mechanisms of appetitive function^5^, ^6^, ^7^ and, more recently, to identify brain function changes associated with weight loss. Food‐cue reactivity has been used to predict various food‐related outcomes including eating patterns (ie, dieting and weight outcomes [ie, gain and loss]).^8^ For example, cross‐sectional research has shown that weight loss is associated with decreases in reward system activation (eg, anterior cingulate cortex and amygdala) in response to visual food cues in individuals who retrospectively report successful loss and maintenance of body weight.^9^ Longitudinal studies comparing brain activation preweight and postweight loss from surgical and a subset of the present behavioral weight management program have also demonstrated decreased activation to appetitive food pictures in regions of the brain previously implicated in food cue reactivity and reward (eg, the parahippocampus, medial prefrontal cortex, insula, and inferior frontal gyrus.^10^, ^11^, ^12^)

Longitudinal studies have also examined predictors of future weight gain and weight loss^7^, ^13^, ^14^, ^15^. In the context of diet, Murdaugh and colleagues^16^ conducted a study including 25 individuals with obesity and found greater preweight loss treatment brain activation to high‐calorie food vs control pictures (cars) in brain regions implicated in reward‐system processes including the nucleus accumbens, anterior cingulate cortex, and insula. Similar correlations with weight loss in brain regions identified in earlier cross‐sectional studies,^17^, ^18^, ^19^, ^20^ including superior occipital cortex, inferior and superior parietal lobule, and prefrontal cortex. Thus, there is evidence that altered activation in reward processing and cognitive control circuitry predicts weight gain 7, 14, 15 and failed weight loss.^16^ Recently, Neseliler and colleagues^21^ examined hormonal and brain activation correlates of weight loss at 1 and 3 months and found that weight loss was correlated with increased activation and functional connectivity in prefrontal cortical regions. These results highlight the importance of prefrontal activity to weight loss. However, brain function does not directly cause weight loss; rather, brain function regulates diet adherence behaviors such as food intake and physical activity (PA) that lead to weight loss. To our knowledge, no studies have yet examined how brain function is linked to the diet adherence behaviors that actually cause weight loss, which is the focus of the current study.

Many published reports have identified treatment adherence as a predictor of weight management success.^3^, ^22^, ^23^ Results from a systematic review by Washburn and colleagues^3^ suggest that optimal weight loss and maintenance are achieved when an intervention consists of both diet and exercise modifications; however, the distinct dieting behaviors and PA modes or amounts associated with success are difficult to specify. Programs that have identified behaviors associated with success, or lack of success, suggest that class attendance, portion‐controlled meals (PCMs, entrees, and shakes), fruit and vegetable consumption, minutes of PA performed, and number of steps taken each week have been associated with successful weight loss and maintenance.^3^, ^22^ Similarly, Carels and colleagues^23^ have identified poor program attendance as being significantly associated with poor weight loss. Despite fundamental differences among diet interventions, it is clear that adherence to a program (ie, class attendance, diet modification, and PA) influences the amount of weight loss and maintained during and at the conclusion of weight management programs.

Research has also linked executive control to health behavior change success and long term adherence.^24^ “Executive control” is defined as the ability to regulate behavior, emotions, and thoughts. It also includes cognitive processes such as inhibition, mental flexibility, working memory, and the ability to plan and execute goal‐oriented actions like health behaviors.^25^ A review by Hall and Marteau^24^ posited that executive control, or behavioral self‐regulation, can influence health status directly and indirectly through health behaviors. Executive control processes, including self‐regulation, are linked to the same prefrontal cortical regions of the brain that have been associated with responses to food cues and cognitive responses to weight loss.^10^, ^16^, ^17^, ^26^, ^27^ Hall and Marteau^18^ propose that there are likely reciprocal effects between executive functioning, self‐regulation, and obesity. For example, poor executive functioning may lead to consistently unhealthy choices, which in turn compromises brain function and further degrades capacity for healthy decision making.^24^, ^26^, ^28^ Similarly, executive control (64) and prefrontal cortex volume has been associated with exercise adherence,^29^ which is often a major component of weight management treatment. This suggests that executive control could play a crucial role in health and adherence behaviors and thereby impact obesity management and treatment.^30^

To date, initial findings suggest the following: (1) Activation in regions of the brain previously implicated in food cue reactivity and cognitive control may predict weight loss success; (2) adherence behaviors such as intervention attendance, diet modification, and PA are also related directly linked to weight loss success; and (3) executive control and its associated brain regions may be associated with behavior change processes that lead to weight loss. What is unknown is the pathway through which brain activity influences behaviors that lead to weight loss. Therefore, the goal of the present exploratory investigation was to identify the contributions of baseline brain activation adherence behavior patterns and the associated connections to weight loss at the conclusion of a 3‐month weight loss intervention.

## 2 MATERIALS AND METHODS

The present investigation is an analysis from the first 3 months (12 weeks) of a 9‐month study. A detailed description of the materials and methods for this study can be found in Szabo‐Reed et al.^31^ This study consisted of a 12‐week diet followed by a 6‐month maintenance period. Functional magnetic resonance imaging (fMRI) scans were completed on participants with obesity (BMI 30 to 45 kg/m^2^) with a visual food cue reactivity fMRI paradigm in a baseline session before participants entered the weight management program. The current analyses focus on fMRI data collected during the baseline scanning session.

### 2.1 Participants

Individuals with obesity (N = 82) were recruited and enrolled in the study; N = 79 completed the 12‐week weight management program; however, only N = 75 had complete data (fMRI, behavioral, and follow‐up) for analyses and were included in all analyses. Baseline demographics and sample characteristics are included in Table 1. All inclusion and exclusion criteria have been previously detailed elsewhere.^31^ Briefly, participants were included in the study if they met the following inclusion criteria: (1) age 21 to 55 years, (2) BMI of ≥30.0 to 45.0 kg/m^2^, and (3) clearance for participation from their primary care physician. Approval for this study was obtained from the Human Subjects Committee at the University of Kansas Medical Center‐Kansas City.

**Table 1:** Demographic and sample characteristics

Variable	Label	N	Mean	SD	Min	Max
Age		75	37.9	8.2	23	55
Sex	Female	75	53 (70.6%)			
Race		75				
	White	52	69.4%			
	Black	20	26.6%			
	Am Ind	1	1.3%			
	Other/unknown	2	2.7%			
Ethnicity		75				
	Hispanic	6	8.0%			
	Non‐Hispanic	64	85.3%			
	Other/unknown	5	6.7%			
IQ (WASI)		75	112.4	11.1	86	132
BMI @ BSL		75	35.4	3.4	30.1	44.0
BMI @ 3 mo		75	31.6	3.4	25.3	40.6
Wt @ BSL		75	99.4	15.1	72.8	136.5
Wt @ 3 mo		75	89.4	13.1	66.4	125.8
%Wt Change		75	−9.92	5.17	+0.41	−23.7
Steps		75	9601	2291.29	3754.59	17445.16
mPA		75	30.55	11.42	12.34	80.65
Shake		75	2.61	.37	1.43	2.98
Entrees		75	1.83	.14	1.31	2.04
Fruit		75	2.71	.3	0.86	7.3
Veg		75	3	.95	1.41	7.01
L‐MFG/DLPFC		75	.18	.22	−0.39	.81
R‐MFG		75	.17	.23	−.42	.60

Abbreviations: BL, baseline; L‐MFG/DLPFC, left middle frontal gyrus/dorsal lateral prefrontal cortex; mPA, average of weekly minutes of physical activity; R‐MFG, right middle frontal gyrus; Wt, weight in kg.

* Significant pathway at p<.05

### 2.2 Assessments

During the fMRI appointment, participants completed two 1‐hour MRI scans (premeal and postmeal), consumed a 500‐kcal meal, and had anthropometric assessments taken. The order of premeal and postmeal scans was counterbalanced.

### 2.3 Anthropometrics (body weight, height, and BMI)

Body weight was recorded at baseline and 3 months using a digital scale accurate to ±0.1 kg (Befour Inc Model #PS6600, Saukville, WI). All participants were weighed after arriving for MRI appointments, at least 4‐hour fasting. Participants weighed in standard hospital scrubs after attempting to void. Height was measured using a stadiometer (Model PE‐WM‐60‐84, Perspective Enterprises, Portage MI), and body mass index (BMI; kg/m^2^) was calculated.

### 2.4 fMRI food cue reactivity paradigm

Participants viewed pictures of food, animals, and blurred low‐level baseline images after fasting at least 4 hours and after consuming a standardized meal.^31^ Food and animal images were obtained from professional stock photography and matched on brightness, resolution, and size. The paradigm used pictures of live animals as control stimuli in order to control for general interest, familiarity, and visual richness so that image groups can be matched for valence and arousal.^17^, ^18^, ^32^ In addition, blurred objects were included in the paradigm as a low‐level baseline comparison. For this, the food and animal images were blurred, so that the objects are not identifiable, by applying the fast Fourier transformation (FFT), removing the phase information, and then applying the inverse FFT in MATLAB (The MathWorks Inc, Natick, MA, http://homepages.inf.ed.ac.uk/rbf/HIPR2/fourier.htm) program. All images were presented one time only to each subject. Validation procedures for these images are outlined in a previous publication.^31^

**Figure 1:**
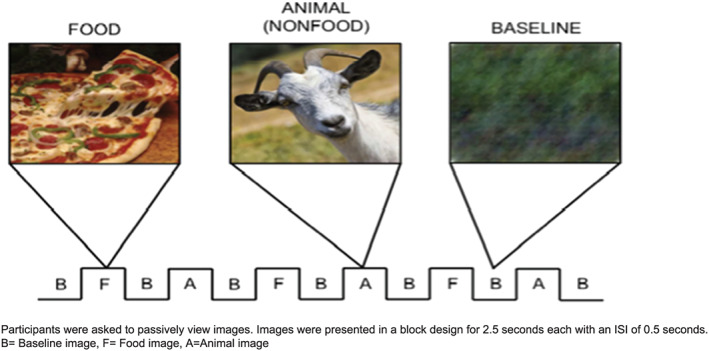
Participants were asked to passively view images. Images were presented in a block design for 2.5 seconds each with an ISI of 0.5 second. A, Animal image; B, baseline image; F, food image

The fMRI scans involved six 30‐second blocks of each stimulus condition type (ie, food and animal), alternated between 30‐second blocks of blurred images. Each block consisted of 10 images. Visual stimuli were presented via a back‐projection system. Stimulus presentation time was 2.5 seconds, with an interstimulus interval (ISI) of 0.5 seconds (see figure 1). The order of category presentation was counterbalanced across subjects using a Latin square design. Participants were instructed to remember as many food and animal images as they could while in the scanner; no responses to stimuli were collected during scanning.

Participants completed a computerized recognition memory task outside the scanner, immediately following the scanning session to ensure they were attentive.

### 2.5 Image acquisition

Scanning was performed in a 3 Tesla head‐only Siemens Allegra scanner (Siemens, Erlangen, Germany) fitted with a quadrature head coil. Participants' heads were immobilized with cushions. Following automated scout image acquisition and shimming procedures performed to optimize field homogeneity, a structural scan was completed. T1‐weighted anatomic images were acquired with a 3D MPRAGE sequence (repetition time/echo time [TR/TE] = 2300/3.06 ms, flip angle = 8°, field of view [FOV] = 192 × 100 mm, matrix = 192 × 192, slice thickness = 1 mm). This scan was used for slice localization for the functional scans, Talairach transformation, and coregistration with fMRI data. Following structural scans, three gradient‐echo blood oxygen level‐dependent (BOLD) scans were acquired in 43 contiguous oblique axial slices at a 40° angle (TR/TE = 3000/30 ms, flip angle = 90°, FOV = 220 mm, matrix = 64 × 64, slice thickness = 3 mm, 0.5 mm skip, in‐plane resolution = 3 × 3 mm, 130 data points). To ensure consistency across subjects and optimize BOLD signal in the ventral and medial portions of the frontal cortex, participants were positioned in the scanner so that the angle of the anterior commissure‐posterior commissure plane was between 17° and 22° in scanner coordinate space.^10^, ^33^ This was verified with a localization scan.

### 2.6 Intervention

Following baseline testing, participants entered the 3‐month weight loss phase of the intervention. Participants attended 60‐minute in‐person, behaviorally based meetings of 5 to 15 individuals that were conducted weekly for 3 months. All meetings used behavioral strategies based on social cognitive theory to promote change in both diet and exercise.^34^, ^35^ Energy intake was reduced to ~1200 to 1500 kcal/day using a combination of commercially available PCMs, fruits and vegetables, low‐calorie shakes, and noncaloric beverages. Participants were provided with a list of selected PCMs and shakes provided by HMR Weight Management Service Corporation (Boston, MA) to select from, fruits and vegetables, and noncaloric beverages that were allowed. Participants consumed a daily minimum of two PCMs (180 to 270 kcal each, provided), at least five servings of fruits and/or vegetables, and three shakes (~100 kcal each, provided). Noncaloric beverages such as diet soda and coffee were allowed ad libitum. When combined with a variety of fruits and vegetables, PCMs (entrees + shakes) provide a diet with all necessary nutrients specified by the Dietary Reference Intakes.^36^ Participants reaching a BMI of 22 kg/m^2^ during the weight loss phase (N = 1) were transitioned to the prevention of weight regain/maintenance diet.

### 2.7 Routine clinic data reports from group meetings

Participants reported the number of PCMs and shakes consumed, the number of fruits and vegetables consumed, minutes of PA completed, and number of steps as recorded on step counters according to their meeting schedule. Participants weighed on a scale at the clinic site at each clinic meeting. At midpoint between meetings, the same information except weight was also collected via toll‐free phone, fax, or email.

### 2.8 Physical activity

Three hundred min/wk of moderately vigorous PA was targeted using a progressive protocol.^37^, ^38^ All exercise was unsupervised. PA was also recorded by pedometer step counts. Participants provided a written record of both PA minutes and steps at each clinic meeting and data collection period. Step counts were used to reinforce and measure lifestyle PA (unplanned or unstructured activity and/or activities of daily living).

### 2.9 Data analysis

#### 2.9.1 General strategy

The goal of the study was to identify brain regions associated with weight loss; thus, brain regions of interest (ROIs) previously cited in the literature (neurosynth) as significantly associated with food cue reactivity and self‐regulation (left and right middle frontal gyrus^39^, ^40^, ^41^, ^42^) were identified. Then how these regions correlated with behaviors that are known to be associated with weight loss was explored.^43^ This approach could provide knowledge regarding the paths by which brain function mediates the actual behaviors that result in weight loss.

### 2.10 fMRI image analysis

#### 2.10.1 Preprocessing and subject‐level analyses

Data preprocessing and statistical analyses were performed in AFNI (Medical College of Wisconsin). Preprocessing steps included motion correction, alignment, spatial smoothing, and spatial normalization. The fMRI images were realigned to the third slice collected in each run to correct for motion. The images were spatially smoothed with a 4‐mm FWHM Gaussian blur. Anatomic images were aligned to functional images and spatially normalized to Talairach stereotaxic space^44^ using AFNI's automated algorithm. Statistical contrasts were conducted using multiple regression analysis with motion parameters included as nuisance regressors. Regressors representing the experimental conditions (ie, food and nonfood) were modeled with a standard hemodynamic response and entered into the multiple‐regression analysis using a random‐effects model.

#### 2.10.2 Group level analysis

Following fMRI data preprocessing and subject‐level analysis, cue‐reactivity ROIs were identified using a whole‐brain voxelwise ANOVA (ie, percent signal change from baseline) to identify brain regions showing a main effect of image type using AFNI's 3dMVM.^45^ Corrections for multiple comparisons were achieved with false discovery rate of *q* < 0.05. Clusters of activation that passed FDR correction and were in a priori regions of the middle frontal gyrus/dorsolateral prefrontal cortex were selected as ROIs based on the role of the dorsolateral prefrontal cortex in self‐regulation and executive function.

### 2.11 Preprocessing behavioral variables

Data were summarized by descriptive statistics including the available number of observations (N), mean, and standard deviation (SD) (see Table 1). Pearson coefficients are provided in Table S1. A factor analysis on the behavioral variables (ie, shakes, entrees, fruit, vegetables, minute of PA, and steps) was conducted; results showed that there exist two factors: PA loading on the minutes of PA (mPA) and steps taken, diet loading on entrees, shakes, and vegetables and fruit (see Table 2). Because variables were measured by different units and had wide variance, we standardized the variables and generated new variables using the arithmetic means of standardized variables. The factor model showed good fit: chi‐square = 31.752, *P* = .201, comparative fit index [CFI] = .951, Tucker‐Lewis index [TLI] = .916, root mean square error of approximation [RMSEA] = .054 (95% CI, 0‐.111), and standardized root mean square residuals [SRMR] = .071 (Table 4).

**Table 2:** Confirmatory factor analysis

	PA				Diet			
	Estimate	SE	Z	*P*(>|*z*|)	Estimate	SE	Z	*P*(>|*z*|)
Steps	1.000							
mPA	.348	.688	.506	.613				
Shake					1.000			
Entrees					.847	.552	1.535	1.125
Fruit					0.220	.212	1.037	.300
Veg					.483	.217	2.226	.026

*Notes.* Chi‐square = 5.604, *P* = .692, CFI = 1.000, TLI = 1.000, RMSEA = .000, SRMR = .052. Results are based on 1000 bootstrap samples.

Abbreviation: mPA, minutes of physical activity.

### 2.12 Structural equation model

The brain regions identified, the factors identified based on the behavior variables, attendance, and weight loss at 3‐month were included in a structural equation model. Our hypothesis was that the behavioral factors (PA and diet) and attendance during the 3‐month intervention period as potential mediators between the brain regions and the 3‐month percent weight loss, with the 3‐month percent weight loss being the dependent variable. The following paths were hypothesized: (1) from brain activation to %attendance, diet, and PA; (2) from %attendance, PA, and diet to 3‐month percent weight loss; (3) from brain activation directly to 3‐month weight loss; and (4) from diet to PA. The ordering of components of the path structure was developed based on chronology sequence (ie, baseline brain scans, adherence behaviors measured during the intervention, and postintervention weight loss) and the presupposition that brain modulates behavior, which in turn influences weight loss response. The path option (3) allowed partial instead of total mediation effects. Nonparametric bootstrapping with 1000 samples was used in estimation. Path analysis was performed with the R lavaan package, version 0.6‐5.^46^

### 2.13 Linear regression

As a final step, the proportion of variance in the path model explained by the brain and behavioral variables, both independently and jointly, was evaluated. To determine this, two linear regression models were performed, with the dependent variable of percentage of weight loss at 3 months. The independent predictors for each model were as follows: (1) %attendance, steps, mPA, PCMs, shakes, fruits, vegetables, left middle frontal gyrus/dorsal lateral prefrontal cortex, and right middle frontal gyrus; (2) %attendance, steps, mPA, entrees, shakes, fruits, and vegetables.

### 2.14 Results

Demographic and sample characteristics are displayed in Table 1. The sample (N = 75) was 37.9 ± 8.2 years old, 70.6% female, and primarily white (69.4%) and non‐Hispanic (85.3%). Average baseline BMI was 35.3 kg/m^2^ ± 3.4. During the 3‐month weight management program, participants lost an average of 22.2 ± 4.3 lbs, took 9601 ± 2291.3 steps per day, completed 30.5 ± 11.4 min/day of PA, and consumed 2.61 ± 0.37 shakes, 1.83 ± 0.14 entrees, 2.71 ± 0.3 fruits, and 3.00 ± 0.95 vegetables per day. Attendance to the weight management program was approximately 83%.

**Figure 2:**
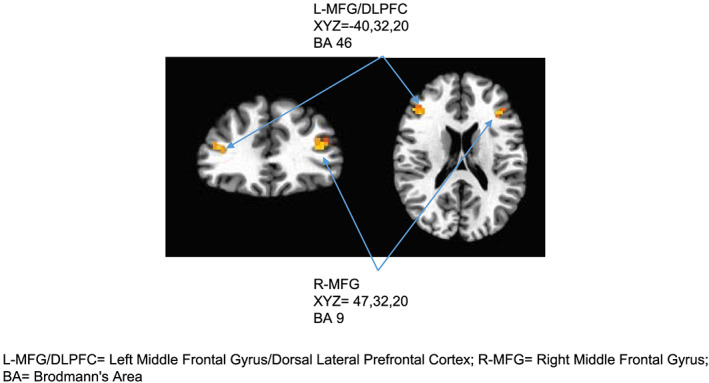
L‐MFG/DLPFC, Left middle frontal gyrus/dorsal lateral prefrontal cortex; R‐MFG, right middle frontal gyrus; BA, Brodmann area

Results of the group level analyses identified regions of the brain that showed a main effect of image type (ie, food and nonfood) (see Figure 2). Specifically, activation in left middle frontal gyrus/dorsal lateral prefrontal cortex (*x*, *y*, *z* = −40, 32, 20, Brodmann area 46, 33 voxels, 8.73 mm^3^) and right middle frontal gyrus (*x*, *y*, *z* = 47, 32, 20, Brodmann area 9, 31 voxels, 8.20 mm^3^) were selected as ROI for this analysis. Previous research has suggested insular activation is associated with food cue reactivity,^47^ while right middle frontal gyrus activation is associated with self‐regulation.^39^, ^40^, ^41^, ^42^ For each of the clusters surviving, this threshold (ie, left middle frontal gyrus and right middle frontal gyrus), the average the percent signal change (food‐animals) across voxels for each subject was created and used as variables to relate to behavioral variables.

Table S1 summarizes the Pearson correlation coefficients using all available observations. Table 3 summarizes results from the structural equation model analysis. The model provided a good fit to the data: chi‐square = 31.752, *P* = .201, CFI = .951, TLI = 0.916, RMSEA = 0.054, and SRMR = .071^48^ (Table 4). The standardized coefficients in the structural equation model can be interpreted as typical linear regression coefficients, eg, for every one standard deviation increase in right middle frontal gyrus brain activation, attendance rate increased by 0.253 of its own standard deviation. Other coefficients are interpreted in the same way.

**Table 3:** Standardized path coefficients

	1000 Bootstrap Samples			
		Std Err	*Z* Value	*P*(>|*Z*|)
%WL3~				
%att	0.451	0.083	5.453	<.0001*
PA	0.418	0.198	2.117	.034*
Diet	0.343	0.200	1.713	.087
L‐MFG/DLPFC	−0.125	0.120	−1.046	.296
R‐MFG	0.158	0.104	1.520	.129
%att~				
L‐MFG/DLPFC	−0.103	0.127	−0.815	.415
R‐MFG	0.237	0.106	2.246	.025*
PA~				
Diet	0.287	0.224	1.279	.201
L‐MFG/DLPFC	0.101	0.125	0.814	.416
R‐MFG	−0.198	0.121	−1.641	.101
Diet~				
L‐MFG/DLPFC	−0.085	0.124	−0.689	.491
R‐MFG	0.082	0.138	0.596	.551
Covariance				
L‐MFG/DLPFC~~R‐MFG	0.331	0.101	3.278	.001*
Indirect effect				
WL_PA_L‐MFG/DLPFC	0.042	.054	0.784	.433
WL_diet_L‐MFG/DLPFC	−0.029	.044	−0.669	.504
WL_att_L‐MFG/DLPFC	−0.047	0.057	−0.825	.409
WL_PA_R‐MFG	−0.083	.061	−1.348	.178
WL_diet_R‐MFG	0.028	.051	0.551	.582
WL_att_R‐MFG	0.107	.050	2.141	.032*
PA_diet_L‐MFG/DLPFC	−0.029	.044	−0.669	.504
PA_diet_R‐MFG	0.024	.047	0.505	.613

Abbreviations: Wt, weight in lbs; BL, baseline; PA, physical activity; L‐MFG/DLPFC, left middle frontal gyrus/dorsal lateral prefrontal cortex; R‐MFG, right middle frontal gyrus.

* Significant pathway at p<.05

**Table 4:** Model goodness of fit

Chi‐square Test		BIC^†^	CFI	TLI	RMSEA				
Statistic	*P* Value				RMSEA	95% CI	*P* (≤.05)	SRMR	
**31.752**	.201	2020	0.951	0.916	0.054	(0, 0.111)	.436	0.071	

Sample‐size adjusted BIC.

**Figure 3:**
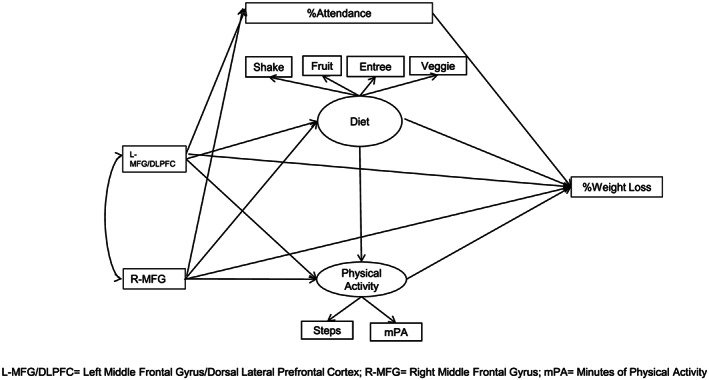
L‐MFG/DLPFC, Left middle frontal gyrus/dorsal lateral prefrontal cortex; R‐MFG, right middle frontal gyrus; mPA, minutes of physical activity

The final model provides a good fit to the data (Table 4). It (Table 3) suggests that^1^ the effect of brain activation on weight loss is mediated by the behavioral variables attendance (see Figure 3). The indirect effect from the right middle frontal gyrus to %attendance to weight loss is significant (*b* = .107, *P* = .032). At the same time, the direct effect from weight loss at 3 months on the left middle frontal gyrus/dorsal lateral prefrontal cortex (*b* = −.125, *P* = .296) and right middle frontal gyrus (*b* = .158, *P* = .129) are not significant.

The independent variance is explained by the behavior (*R* ^2^ = .47), and combined variance (*R* ^2^ = .48) is explained by having both brain and behavior included in the model. These findings suggest that 47% of the variance in the model is uniquely explained by the behavioral variables, and 48% is explained by both the behavioral and brain variables combined.

## 3 DISCUSSION

Results from this study indicate that baseline activation to appetizing food pictures in the left middle frontal gyrus and right middle frontal gyrus predict future weight loss during a weight management intervention. This finding is consistent with previous work implicating the role of the prefrontal cortex in control and self‐control processes needed to regulate eating behavior^39^, ^41^, ^42^ and to lose weight.^16^, ^21^, ^49^ This research is, to our knowledge, the first to model connections between brain activation and adherence behaviors leading to weight loss. The path analysis indicated that activation in left middle frontal gyrus/dorsal lateral prefrontal cortex and right middle frontal gyrus were directly related to weight loss and impacted weight loss via effects on intervention attendance.

Similar to previous research, positive correlations were observed between activation to high‐calorie food vs control images and subsequent weight change in the right middle frontal gyrus (BA 9).^16^, ^50^ The right middle frontal gyrus (BA 9) has also been linked to dietary self‐control and attention to health cues.^51^, ^52^ As observed in the present study, activation in the right middle frontal gyrus (BA 8) has also been predictive of future weight loss.^16^ In summary and combined with previous research, our observations here suggest that the right middle frontal gyrus may play an important role in the self‐regulatory processes, which are necessary for weight loss success and adherence to weight management interventions.

Activation in the left dorsal lateral prefrontal cortex (BA 46) has been previously implicated as a potentially crucial component in diet success.^5^, ^21^, ^42^ The present investigation, as well as others,^11^, ^18^, ^32^ has found similar findings in food vs nonfood picture contrasts. This consistent finding highlights the dorsal lateral prefrontal cortex's role as a behavioral control area as is often found to be chronically activated in overweight and obese individuals, presumably reflecting compensatory processes used to regulate eating behavior^53^, ^54^ and appetite hormones.^21^ Schmidt and colleagues^42^ have also found that individuals with more gray matter volume in the dorsal lateral prefrontal cortex (BA 46) are better at exercising dietary control. Dorsal lateral prefrontal cortex (BA 46) also plays a role in complex cognition including executive control, attention, and inhibitory control. This region has a well‐established role in goal‐directed behavior, specifically when it comes to conflict and self‐monitoring, error detection, executive control, and decision making about risk and reward.^55^ Specifically, increased activation in the dorsal lateral prefrontal cortex has been displayed in obese as compared with healthy weight individuals when viewing food as compared with nonfood stimuli in an fMRI.^18^ In this study, the effects of dorsal lateral prefrontal cortex on weight loss were mediated by attendance to the behavioral meetings associated with the intervention and with PA compliance.

Executive control functions, such as self‐regulation, which include planning and decision‐making that are carried out in prefrontal cortex, have been previously linked to health behavior adherence and change.^24^, ^25^ In this study, greater activation of the right middle frontal gyrus and left middle frontal gyrus/dorsal lateral prefrontal cortex during the food cue reactivity paradigm was associated with attending more intervention classes and completing more steps and minutes of PA and resulted in a greater percentage of weight loss at the end of a 3‐month weight loss intervention consisting of diet and exercise. These findings support the theory that areas of the brain associated with executive control (ie, anterior and dorsolateral prefrontal cortices) may be related to health behavior change (ie, diet and PA), which in turn leads to a change in health status (ie, weight loss).^24^ Obesity has long been associated with decrements in executive control in both adults and children.^56^, ^57^ In addition, reduced executive control abilities have also been linked to the consumption of unhealthy foods and other food‐related choices.^26^, ^27^, ^58^ This includes inhibition (ie, ability to not respond to a stimulus or ignore a palatable food) and planning goal‐oriented behaviors (ie, planning exercises sessions or healthy meals), two behaviors that are important for weight management. Also, a recent study conducted by Mokhtari and colleagues^59^ utilizing a machine learning technique further implicated the dorsal lateral prefrontal cortex and other executive control areas of the brain as important for predicting weight loss success. Similarly, executive control (64) and prefrontal cortex volume has been associated with exercise adherence,^29^ which is often a major component of weight management treatment. Therefore, improving executive control could be one potential avenue for also improving health behavior change and adherence.

Other well‐established relationships between behavior and weight status were also present in this study in addition to the unique brain and behavior relationships. Our model clearly shows a direct relationship between both diet (ie, entrees and shakes consumed) and PA (ie, number of steps and minutes). Such behaviors have also been established to be important to success in other weight management studies as those who “do more” (ie, consume more entrees/shakes or take more steps) are shown to be more successful long term compared with those who “do less”.^3^, ^22^ Szabo‐Reed and colleagues^22^ also established that attendance to the behavioral intervention classes is also associated with initial weight loss and long‐term weight management success. Therefore, the current findings are consistent with previous work and significantly extend it by providing a key baseline measure, brain activation, which may be a useful tool in the future for predicting weight loss and weight management success.

### 3.1 Limitations

There are several limitations associated with this study. First, the information obtained from the weekly clinic data reports regarding diet and PA behaviors during the weight loss intervention were self‐reported by the participants. Therefore, errors or misreporting may have occurred. Future attempts to collect such data should employ automatic and objective data collection methods when possible (ie, accelerometers and Fitbit). There may exist other viable path structures/models to fit our observed data. This model was selected for the current work because of interpretability and study time course. The sample size in the study is small as these are results from an exploratory analysis. This exploration was completed to provide valuable empirical data for future studies. Nonetheless, findings from this study should be considered preliminary until they are validated in an independent sample.

## 4 CONCLUSIONS

The present study represents a first attempt to establish pathways between baseline brain activation, weight management adherence, and weight loss. These findings present exploratory outcomes that suggest that the effects of baseline brain activation associated with food cue reactivity related to self‐regulation in the left middle frontal gyrus/dorsal lateral prefrontal cortex and right middle frontal gyrus are expressed directly and through effects on class attendance. Prefrontal cortex‐mediated executive control and self‐regulation have been established as a key contributor to change and maintenance of health behaviors, especially when it comes to planning and healthy behavior decision‐making. Although results from this study should be considered exploratory and preliminary, findings support these connections and are consistent with growing evidence of the importance of prefrontal cortex activity on the regulation of eating behavior.^21^ In conclusion, this research indicates that prefrontal cortical activation influences health behavior change in the context of an intervention to produce weight loss and manage obesity. More research is needed to determine whether such brain‐behavior interactions can be modified (eg, tDCS^60^) to increase weight loss success and how such interventions can aid in managing the current obesity epidemic.

## FUNDING INFORMATION

Funding is provided by R01‐DK080090 (Savage) and KL2‐TR002367 (Szabo‐Reed); HMR Weight Management Service Corp, Hoglund Brain Imaging Center, is supported by a generous gift from Forrest and Sally Hoglund and funding from the National Institutes of Health (UL1‐TR000001).

## Supporting information

Table S1. Pearson correlation coefficients and sample size, mean, and SD
