# Supplementary material for: Modeling interactions between brain function, diet adherence behaviors, and weight loss success
Source: Obes Sci Pract. 2020 Feb 25;6(3):282–92. doi: 10.1002/osp4.403 (PMC7278911; doi:10.1002/osp4.403)
Supplement: Supplementary file 1 — Table S1. Pearson correlation coefficients and sample size, mean, and SD [file OSP4-6-282-s001.pptx]

## Slide 1
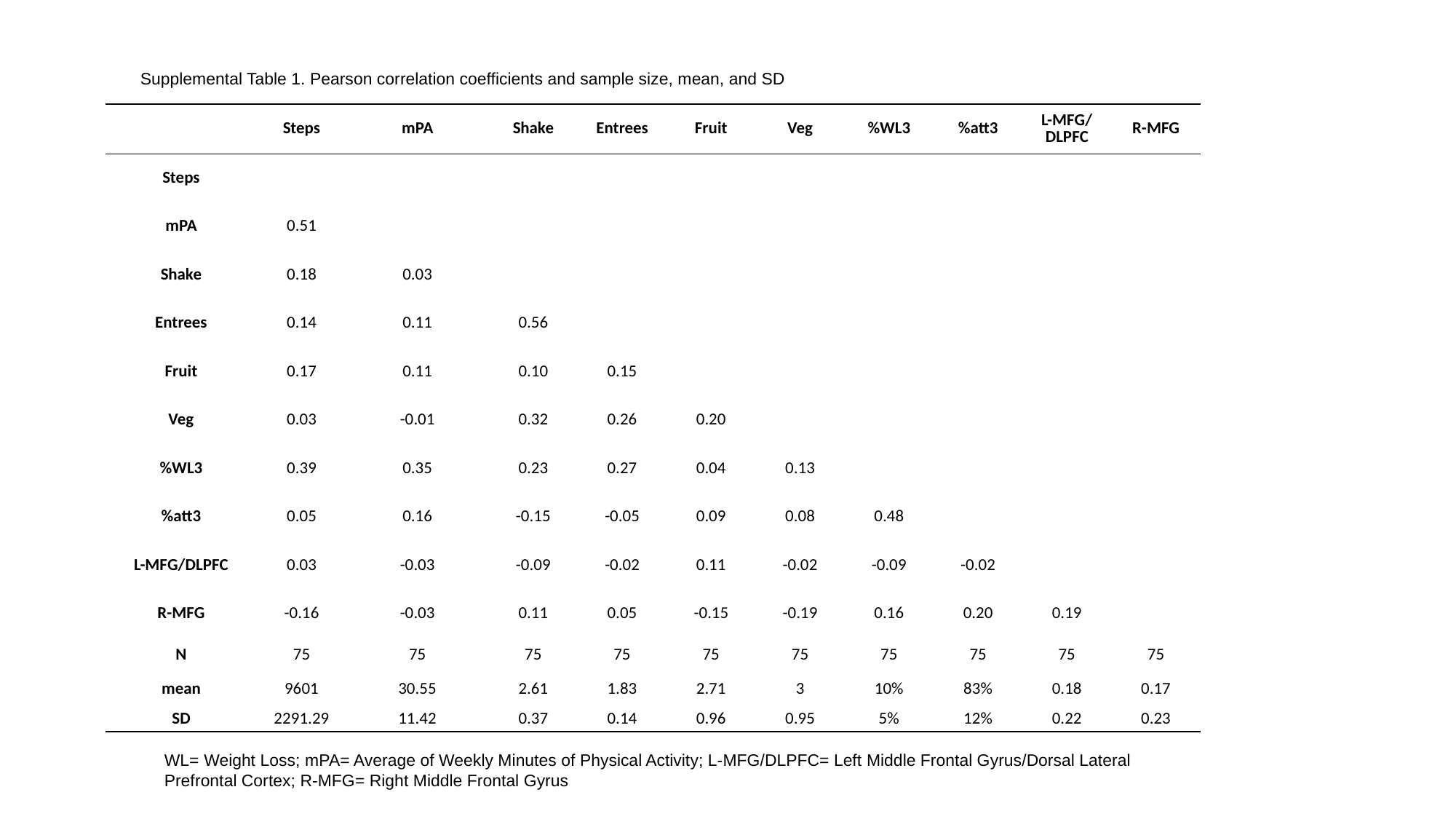

Supplemental Table 1. Pearson correlation coefficients and sample size, mean, and SD
| | Steps | mPA | Shake | Entrees | Fruit | Veg | %WL3 | %att3 | L-MFG/DLPFC | R-MFG |
| --- | --- | --- | --- | --- | --- | --- | --- | --- | --- | --- |
| Steps | | | | | | | | | | |
| mPA | 0.51 | | | | | | | | | |
| Shake | 0.18 | 0.03 | | | | | | | | |
| Entrees | 0.14 | 0.11 | 0.56 | | | | | | | |
| Fruit | 0.17 | 0.11 | 0.10 | 0.15 | | | | | | |
| Veg | 0.03 | -0.01 | 0.32 | 0.26 | 0.20 | | | | | |
| %WL3 | 0.39 | 0.35 | 0.23 | 0.27 | 0.04 | 0.13 | | | | |
| %att3 | 0.05 | 0.16 | -0.15 | -0.05 | 0.09 | 0.08 | 0.48 | | | |
| L-MFG/DLPFC | 0.03 | -0.03 | -0.09 | -0.02 | 0.11 | -0.02 | -0.09 | -0.02 | | |
| R-MFG | -0.16 | -0.03 | 0.11 | 0.05 | -0.15 | -0.19 | 0.16 | 0.20 | 0.19 | |
| N | 75 | 75 | 75 | 75 | 75 | 75 | 75 | 75 | 75 | 75 |
| mean | 9601 | 30.55 | 2.61 | 1.83 | 2.71 | 3 | 10% | 83% | 0.18 | 0.17 |
| SD | 2291.29 | 11.42 | 0.37 | 0.14 | 0.96 | 0.95 | 5% | 12% | 0.22 | 0.23 |
WL= Weight Loss; mPA= Average of Weekly Minutes of Physical Activity; L-MFG/DLPFC= Left Middle Frontal Gyrus/Dorsal Lateral Prefrontal Cortex; R-MFG= Right Middle Frontal Gyrus
